# Demographic profile of oral nonodontogenic cysts in a Brazilian population

**DOI:** 10.4317/medoral.19335

**Published:** 2013-12-07

**Authors:** Ana C. Uchoa-Vasconcelos, Daniel J. Filizola-de Oliveira, Stephanie J. Roman-Martelli, Adriana Etges, Ana P. Neutzling-Gomes, Sandra B. Chaves-Tarquínio

**Affiliations:** 1 Pos-Doc Fellowship, Post-graduate Program in Dentistry, Dental School, Federal University of Pelotas - Brazil; 2 Undergraduate Student, Dental School, Federal University of Pelotas - Brazil; 3 Ph.D., Post-graduate Program in Dentistry, Department of Oral Pathology & Stomatology, Dental School, Federal University of Pelotas - Brazil

## Abstract

Objectives: The purpose of this study was to determine the clinical and demographic characteristics of oral non-odontogenic cysts (ONOC) in a Brazilian population over a 53-year period and to compare this data with the literature. Study Design: A total of 20.391 biopsies records were evaluated, from April/1959 to August/2012. Cases of oral developmental cysts were selected. Data regarding age, gender, time of evolution, and anatomic site of all cases were collected. The data were analyzed by descriptive statistics. Results: Among 20.391 oral biopsies, 71 (0.35%) met the criteria of ONOC. Females accounted for 50.70% of all cases. The mean age observed was 38.14 years (range: 5-88 years). Nasopalatine duct cysts, oral lymphoepithelial cysts and epidermoid cysts were the most common ONOC, accounting for 63 cases (88.73%). Nasopalatine duct cysts occurred in 31 cases (43.66%), followed by 22 patients with oral lymphoepithelial cysts (30.99%) and 10 cases of epidermoid cysts (14.08%). Nasopalatine duct cysts revealed predominance among males (58.06). Oral lymphoepithelial cysts were more commonly observed in tongue (50%). Epidermoid cysts were most frequently found in the buccal mucosa (40.00%). Conclusions: The differential diagnosis of ONOC is based on the clinical, radiological, and histological findings. It is difficult to establish an epidemiological profile of ONOCs, considering the low frequency of these lesions and the divergences in the demographic and clinical presentation data among different populations.

** Key words:**Diagnosis, epidemiology, jaw cysts, mouth.

## Introduction

Cyst represents a pathologic cavity surrounded by epithelium, with a fluid or semisolid material in its interior (1). Despite this definition, there are cysts of the oral and maxillofacial region that are not lined by epithelium, such as the mucous extravasation cysts of the salivary glands, the antral pseudocyst, the aneurismal bone cyst and the solitary bone cyst. Most authors prefer to describe those pathological cavities as pseudocysts (2). Nonodontogenic cysts (NOC) are a diverse group of lesions whose possible etiopathogenesis has been discussed over the years and there is a general consensus about their possible origin and development of most of them. This group includes nasolabial cyst, nasopalatine duct cyst, incisive papilla cyst, oral lymphoepithelial cyst, thyroglossal duct cyst, palatine cyst of newborn, epidermoid cyst and dermoid cyst (3).

Nasopalatine duct cyst, oral lymphoepithelial cyst and intraoral epidermoid cyst are the three most common NOCs (4). The nasopalatine duct cyst is believed to arise from epithelial remnants of the nasopalatine duct, which connects the nasal and oral cavities in the developing fetus (5-7). The oral lymphoepithelial cyst may be located in ectopic foci of embriologic epithelium within lymphoid tissue or being related to the obstruction of crypts of normal oral tonsils. Some authors believe that it is also possible that epithelial cells could be traumatically implanted into deeper tissues leading to the cystic formation in other areas of the oral region (8). The epidermoid cysts that appear in the midline floor of mouth are believed to be a result of entrapped ectodermal tissue of the first and second branchial arches (9).

These lesions are rare, asymptomatic, and tend to grow slowly, possibly due to an increase in luminal hydrostatic pressure (3,5). The histologic, clinical and radiographic characteristics of some of them are the criteria used for the differential diagnosis among NOCs.

Despite the large number of reports of NOC, there is little information on the relative incidence and the demographic profile of these lesions in different populations (3). The aim of this study is to present a survey of 71 cases of oral nonodontogenic cysts (ONOC) diagnosed at an Oral Pathology Service in a Brazilian population during a 53-year period, including their clinical and demographic characteristics. Furthermore, we also discuss the results comparing them with literature data.

## Material and Methods

This study was approved by the Ethics Committee of Federal University of Pelotas (UFPel-77/12). A total of 20.391 biopsies records of the Center of Diagnosis of Oral Diseases (CDOD) - Federal University of Pelotas (UFPel), Brazil, were evaluated, from April/1959 to August/2012. Cases of oral development cysts including nasolabial cyst, nasopalatine duct cyst, oral lymphoepithelial cyst, thyroglossal duct cyst, palatine cysts of newborn, incisive papilla cyst, epidermoid and dermoid cysts were analyzed. The criteria used to include the ONOC in this study followed the one proposed by Nonaka *et al.* (3), excluding those cysts from an extra oral location. Data regarding age, gender, time of evolution, and anatomic site of all samples were collected. The data were analyzed by descriptive statistics, using the SPSS 17.0 software (Statistical Package for the Social Sciences, Chicago, IL, USA).

## Results

Among 20.391 oral biopsies from the CDOD – Federal University of Pelotas, Brazil, since 1959 to 2012, seventy-one (0.35%) met the criteria of ONOC. Of these, 36 cases (50.70%) occurred in females. The mean age at diagnosis was 38.14 years (range: 5-88 years). Nasopalatine duct cysts, oral lymphoepithelial cysts and epidermoid cysts were the most common ONOC, accounting for 63 cases (88.73%). Nasopalatine duct cysts occurred in 31 cases (43.66%), followed by 22 patients with oral lymphoepithelial cysts (30.99%) and 10 cases of epidermoid cysts (14.08%). Taken together these most common cysts were more prevalent in males (50.79%) and the mean age was 40.14 years (range: 14-88 years). Nasopalatine duct cysts revealed predominance among males (58.06%). Oral lymphoepithelial cysts were more commonly observed in tongue (50%). Epidermoid cysts were most frequently observed in the buccal mucosa (40%). The other cysts corresponded to 11.27% of all NOC, and included nasolabial cysts and dermoid cysts. Intraoral thyroglossal duct cyst and palatal cyst of newborn were not found. The unique case of incisive papilla cyst was taken together to the nasopalatine duct cyst, because they are related lesions that are located within and outside the nasopalatine channel ( Table 1, Table 2).

**Table 1:**
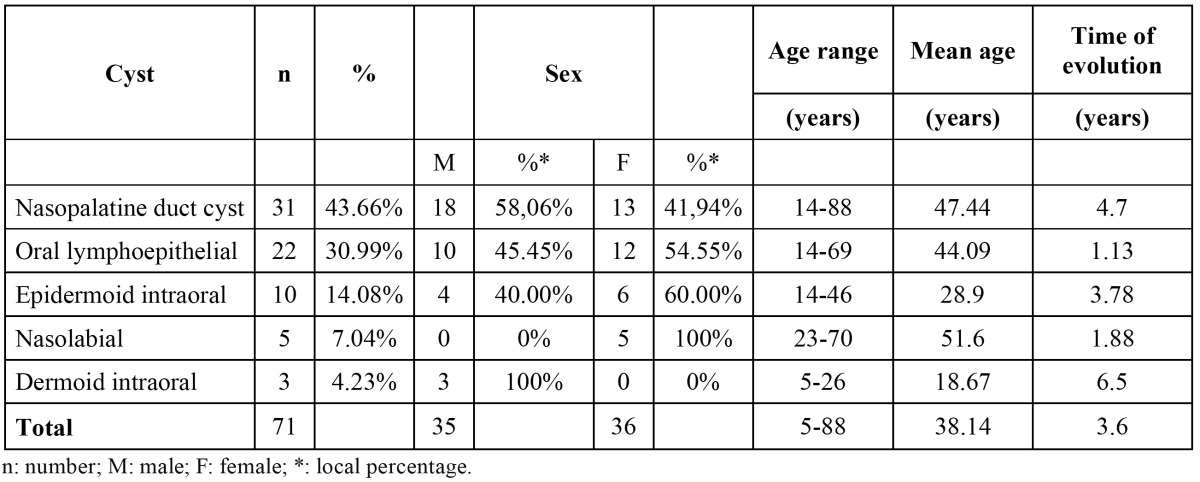
Demographic and clinical characteristics of development cysts.

**Table 2:**
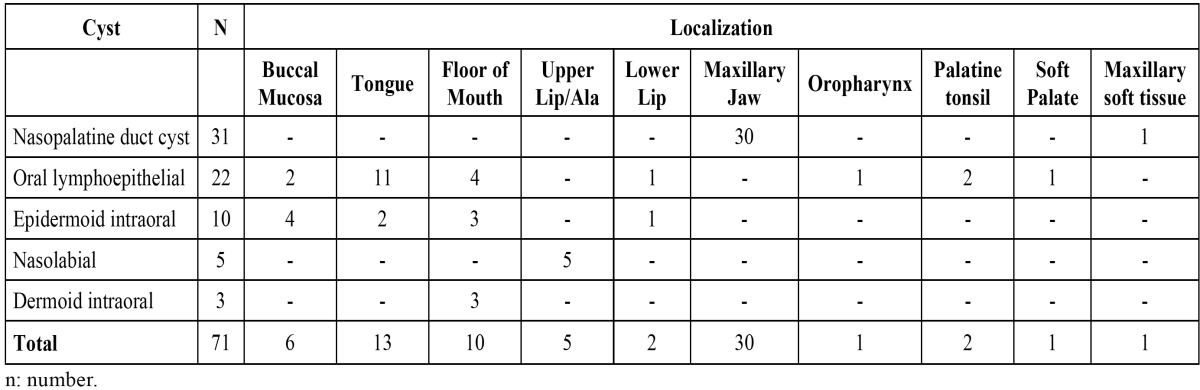
Localization of nonodontogenic cysts by site.

## Discussion

The term NOC includes a varied group of lesions that affect the oral and maxillofacial region, such as nasolabial cyst, nasopalatine duct cyst, incisive papilla cyst, oral lymphoepithelial cyst, thyroglossal duct cyst, palatal cyst of newborn, epidermoid and dermoid cysts (3). The histogenesis, relative frequency of occurrence, clinical features, and biological behavior vary among these lesions (1). In the present study, a relatively low frequency (0.35%) of ONOC was observed from all biopsies evaluated in a 53 year period. This figure is lower than that observed by Daley *et al.* (4), which showed a frequency of 1.0% of 40.000 biopsies. However, in the mentioned study they might have included extra oral cysts, which could explain the different frequencies observed in both surveys.

In the present investigation, ONOC were similarly distributed among females/males (50.70%/49.30%). This finding represents a small difference from the prevalence observed by Nonaka *et al.* (3) who found a female/male ratio of 1.9:1, when investigated the frequency of NOC in another Brazilian study. This might be explained by the difference in the prevalence of each NOC, causing a variation in gender predominance. The exclusion of extra oral NOC in our study also could be plausible reason of these findings. Concerning to age range, the present research revealed a peak of incidence in the fourth decade, similar to the findings reported by Nonaka *et al.* (3) and Grossman *et al.* (1), that demonstrated larger number of NOC cases.

Nasopalatine duct cyst, oral lymphoepithelial cyst, and intraoral epidermoid cyst, were the most frequent ONOCs, accounting for 88.73% of all cases raised in this study. Another ONOCs were observed, such as nasolabial cyst and intraoral dermoid cyst.

Nasopalatine duct cyst have been described to be the most common NOC of the oral cavity (1,3-7,10), with frequencies ranging from 68.8 to 73.2% (3). In this study, this lesion was the most prevalent ONOC, with a frequency of 43.66%, lower than the data reported in the literature (3). This lesion showed predilection for men, similar to the findings observed by others (1,5,7,10,11). The diagnosis of nasopalatine duct cyst usually occurs between the fourth and sixth decades of life (3,10), which was also observed in this study, with a mean age of 47.44 years old. One of thirty-one cases represented an incisive papilla cyst and was considered together with the other nasopalatine duct cysts. Nowadays, most authors consider that both are related lesions, being the incisive papilla cyst the one located outside the nasopalatine channel (10).

The oral lymphoepithelial cyst showed a frequency of 30.99%, being the second most prevalent ONOC in this study, in agreement with the literature (4). It is considered a rare lesion, corresponding from 0.09 to 0.18% of all lesions diagnosed at other oral pathology services (3). Regarding gender, the present study revealed a slight predilection for females (54.44%). Some reported series show a female predominance (3,8), while others demonstrate that males are more affected (12,13). The oral lymphoepithelial cyst may occur in all ages, but it is most frequently reported between the third and fourth decades of life (8,13). In the present study the mean age was 44.09 years old, similar to the data of other reports (3,8). In relation to the site of occurrence, the literature reports the floor of the mouth as the most common, followed by the tongue (3,8,12-14). However, our data shows the tongue as the most prevalent site, and the floor of the mouth as the second one. It is important to take in mind that oral lymphoepithelial cyst may occur wherever normal or accessory lymphoid tissue is present (8), like the floor of the mouth, foliaceous papillae and lateral borders of the tongue. The pathogenesis of this cyst is uncertain. Some authors have hypothesized that ectopic foci of embriologic epithelium become entrapped within lymphoid tissue and may proliferate to form a cyst (15). However, other authors have suggested that oral lymphoepithelial cysts are the result of obstruction of crypts of otherwise normal oral tonsils (13,15). It is also possible that traumatic implantation of epithelial cells into deeper tissues can lead to cyst formation (13). This condition must be differentiated from lesions that appear as submucosal papules or nodules such as lipoma and mucoceles. Sialolithiases and minor salivary gland tumors must also be included in clinical differential diagnosis (8,13,15). Histopathologically, it presents as a central cystic lesion lined with stratified squamous epitelium surrounded by lymphoid tissue similar to the histopathological findings in cervical lymphoepithelial cyst (15).

The intraoral epidermoid cyst was the third most prevalent in this case series, accounting for 14.08% of the ONOC, which is in accordance with a previous study (4). It is reported in the literature that epidermoid cyst affects predominantly males (16,17). In contrast, the present study showed a slight predilection for females. Most patients with intraoral epidermoid cyst are in the range between 10 and 35 years of age (3,9), which is in accordance with our findings that present a mean age of 28.9 years. The floor of the mouth is considered the most common site of occurrence (3,9,17). Nevertheless, in this study the buccal mucosa was the most common site, followed by the floor of the mouth.

Other cysts were observed in the present case series, with lower frequencies than the previously mentioned cysts. Nasolabial cyst is a rare NOC, and presented a frequency of 7.04%, similar to the findings of another study (3). It affected only females, and the mean age was 51.6 years old, both data are in accordance with the literature (4). The intraoral dermoid cyst showed a frequency of 4.23%, which is significantly lower than the data reported by Nonaka *et al.* (3), what might be explained by the exclusion of the extra oral dermoid cyst in our study. Although there is no predominant gender according to the recent literature (18), there is some evidence showing a male predominance (19). The current study confirmed this male tendency of occurrence, because only this gender was affected. All these cysts were located in the floor of the mouth. The age range (18.7 years old) was in accordance with the previously reported data, which indicates that the age of most patients with these lesions range between the second and third decades of life (18).

The demographic and clinical characteristics of some of the cysts presented by the studied population were different than those reported in the literature. Nasopalatine duct cysts, oral lymphoepithelial cysts and intraoral epidermoid cysts were the most frequent ONOCs found in this case series. Considering the divergences in the demographic and clinical presentation data among different populations and the low frequency of NOCs, more studies with larger series are necessary to establish an epidemiological profile of these lesions. In addition, there is a lack of studies regarding exclusively the ONOCs, which results in little information about the relative incidence of these lesions.

It is noteworthy to mention that retrospective studies of lesions occurrence and characteristics from the files of diagnostic services is a useful and popular tool in the field of Oral Pathology and Stomatology (20). The study of conditions with very low prevalence, like the ONOCs, is difficult to be carried out with another study design.
